# Automatic tumor segmentation and metachronous single-organ metastasis prediction of nasopharyngeal carcinoma patients based on multi-sequence magnetic resonance imaging

**DOI:** 10.3389/fonc.2023.953893

**Published:** 2023-03-28

**Authors:** Yecai Huang, Yuxin Zhu, Qiang Yang, Yangkun Luo, Peng Zhang, Xuegang Yang, Jing Ren, Yazhou Ren, Jinyi Lang, Guohui Xu

**Affiliations:** ^1^ School of Medicine, University of Electronic Science and Technology of China, Chengdu, China; ^2^ Department of Radiation Oncology, Radiation Oncology Key Laboratory of Sichuan Province, Sichuan Clinical Research Center for Cancer, Sichuan Cancer Hospital & Institute, Sichuan Cancer Center, Affiliated Cancer Hospital of University of Electronic Science and Technology of China, Chengdu, China; ^3^ Applied Nuclear Technology in Geosciences Key Laboratory of Sichuan Province, Chengdu University of Technology, Chengdu, China; ^4^ School of Computer Science and Engineering, University of Electronic Science and Technology of China, Chengdu, China; ^5^ Department of Interventional Radiology, Sichuan Clinical Research Center for Cancer, Sichuan Cancer Hospital & Institute, Sichuan Cancer Center, Affiliated Cancer Hospital of University of Electronic Science and Technology of China, Chengdu, China; ^6^ Department of Radiology, Sichuan Clinical Research Center for Cancer, Sichuan Cancer Hospital & Institute, Sichuan Cancer Center, Affiliated Cancer Hospital of University of Electronic Science and Technology of China, Chengdu, China

**Keywords:** nasopharyngeal carcinoma, metachronous single-organ metastases prediction, multimodal magnetic resonance imaging, automatic learning, intelligent prediction

## Abstract

**Background:**

Distant metastases is the main failure mode of nasopharyngeal carcinoma. However, early prediction of distant metastases in NPC is extremely challenging. Deep learning has made great progress in recent years. Relying on the rich data features of radiomics and the advantages of deep learning in image representation and intelligent learning, this study intends to explore and construct the metachronous single-organ metastases (MSOM) based on multimodal magnetic resonance imaging.

**Patients and methods:**

The magnetic resonance imaging data of 186 patients with nasopharyngeal carcinoma before treatment were collected, and the gross tumor volume (GTV) and metastatic lymph nodes (GTVln) prior to treatment were defined on T1WI, T2WI, and CE-T1WI. After image normalization, the deep learning platform Python (version 3.9.12) was used in Ubuntu 20.04.1 LTS to construct automatic tumor detection and the MSOM prediction model.

**Results:**

There were 85 of 186 patients who had MSOM (including 32 liver metastases, 25 lung metastases, and 28 bone metastases). The median time to MSOM was 13 months after treatment (7–36 months). The patients were randomly assigned to the training set (N = 140) and validation set (N = 46). By comparison, we found that the overall performance of the automatic tumor detection model based on CE-T1WI was the best (6). The performance of automatic detection for primary tumor (GTV) and lymph node gross tumor volume (GTVln) based on the CE-T1WI model was better than that of models based on T1WI and T2WI (AP@0.5 is 59.6 and 55.6). The prediction model based on CE-T1WI for MSOM prediction achieved the best overall performance, and it obtained the largest AUC value (AUC = 0.733) in the validation set. The precision, recall, precision, and AUC of the prediction model based on CE-T1WI are 0.727, 0.533, 0.730, and 0.733 (95% CI 0.557–0.909), respectively. When clinical data were added to the deep learning prediction model, a better performance of the model could be obtained; the AUC of the integrated model based on T2WI, T1WI, and CE-T1WI were 0.719, 0.738, and 0.775, respectively. By comparing the 3-year survival of high-risk and low-risk patients based on the fusion model, we found that the 3-year DMFS of low and high MSOM risk patients were 95% and 11.4%, respectively (p < 0.001).

**Conclusion:**

The intelligent prediction model based on magnetic resonance imaging alone or combined with clinical data achieves excellent performance in automatic tumor detection and MSOM prediction for NPC patients and is worthy of clinical application.

## Introduction

Nasopharyngeal carcinoma (NPC) is a common head and neck cancer in South China, and 47.7% of new cases worldwide have been reported in China (1). With the application of intensity-modulated therapy technology and advances in comprehensive treatment, the 5-year overall survival of NPC reached more than 80% (2). However, distant metastasis is still its main failure mode (3), largely due to the fact that early distant metastasis prediction for NPC patients is quite elusive. This poses an obstacle to early intervention for those patients at high risk of distant metastases.

Distant metastases fall into different categories. For instance, metachronous single-organ metastases (MSOM) refer to the cases where patients suffer from single-organ (e.g., liver, lung, or bone) metastases more than 6 months after treatment. This is also termed as oligometastases, which contrasts with multiple metastases. Different categories of metastases have been reported to differ in their 5-year overall survival. For example, the 5-year overall survival of NPC patients with metachronous liver metastases is 28.6% (4). Jeremy Chee et al. showed that the median survival time of NPC patients with oligometastases was 24.8 months, whereas that of patients with multiple metastases was only 12.8 months (5). With the emergence of multiorgan metastases, the patients’ condition will deteriorate rapidly. Considering the negative impact of metastases on the patients’ survival, there is a need to improve accuracy for pretreatment prediction of single-organ metastases for patients, when a high risk of distant recurrence is present. Once correctly predicted, some aggressive treatment strategies could be applied during treatment so as to achieve a better prognosis.

However, no recognized distant metastasis prediction marker or system of NPC could be found until now. To solve the problem, researchers in the world had explored the gene expression and radiomics-based signature to predict distant metastases of NPC. The 13-gene-based signature reported by Xin-Ran Tang et al. showed a C index of 0.725 in an internal validation cohort to predict distant metastasis-free survival (DMFS) (6). As the accessibility of the gene test restricted the clinical application, other researchers tried to construct a distant metastasis prediction model based on MRI data prior to treatment (7). To establish prognostic or predictive models is the main application area of radiomics (8). Accurate prediction of disease outcome is of great significance for guiding tumor treatment and prognosis judgment.

Radiomics transforms medical image data into high-throughput characterization data that can be automatically acquired (9). Using a radiomics platform, omics information of intratumor heterogeneity can be obtained from a huge amount of imaging data, which are often related to tumor stages, prognosis, and treatment responses (10). Studies have confirmed that radiomic parameters are associated with progression-free survival and treatment response in patients with nasopharyngeal carcinoma (11, 12), and some researchers have developed and validated magnetic resonance imaging-based radiomics to predict distant metastases of nasopharyngeal carcinoma based on traditional radiomic methods (7). Radiomics requires convenient, intelligent, and fast analysis and processing of large amounts of data. However, with its natural drawback of low automation and standardization, as well as cumbersome and time-consuming feature extraction, the traditional radiomic showed relatively low accuracy and robustness for prediction.

To overcome the drawback of traditional radiomics, deep learning can be of great help. Some researchers developed deep learning radiomics (DLR), which showed potential clinical application value in improving the accuracy and reliability of the diagnostic and predictive value of radiomics. Deep learning is a concept of artificial neural network research in machine learning. Some researchers applied deep learning to predict lung cancer gene mutations based on the histopathological morphology of lung cancer (13); another study explored the value of radiomics features to predict the efficacy of neoadjuvant chemoradiotherapy for locally advanced rectal cancer (14), and some other researchers used deep learning and performed dual-energy CT radiomics to predict lymph node metastases in gastric cancer (15). Deep learning has natural advantages in treatment response evaluation and prognosis prediction. Although automatic segmentation of nasopharyngeal carcinoma based on deep learning was usually applied in diagnosis and radiotherapy practice (16–19), no one explored the metachronous single-organ metastases prediction model based on DLR until now.

In spite of the obvious advantages, this method based on deep learning to detect nasopharyngeal carcinoma and predict metachronous single-organ metastases still faces a “reproducibility/replicability” crisis, with a large amount of basic and preclinical research not being reproducible. The previous work extracted the slice with the largest tumor area for one patient as input image sample, and they constructed a single deep feature extraction model to predict DMFS (20). Although it was simple, it suffered from low performance when there were limited data. To overcome this issue, we proposed a novel two-stage framework based on transfer learning to make prediction for single-organ metastases of NPC. In the first stage, a detection model was trained on the train set. The aim of this stage was to pretrain the feature extraction model. In the second stage, a feature extraction model was fine-tuned to make prediction for single-organ metastases. The parameters of this feature extraction model were initialized from the feature extraction part of the detection model trained in the first stage. Experiment results showed that our methods outperformed the comparison method (20) in the T1WI, T2WI, and CE-T1WI sequences.

In addition, we proposed an early fusion multimodal prediction model to combine the clinical data and MRI sequences. Experiment results showed that adding the clinical data improved the performance of the prediction model in the T1WI, T2WI, and CE-T1WI sequences. The AUC was improved by 1.6%, 2.4%, and 4.2% respectively in the T1WI, T2WI, and CE-T1WI sequences. This radiomics deep-learning based platform we developed on the basis of multisequence magnetic resonance imaging is an automatic tumor detection and segmentation approach, to detect MSOM of NPC.

There are two contributions of the paper. Firstly, we proposed a two-stage model based on transfer learning to predict MSOM of NPC. In addition, we proposed an early fusion model to combine the clinical data with MRI sequences to predict MSOM of NPC. This study provided a new insight to predict metachronous single-organ metastases prior to treatment, which could automatically detect the nasopharyngeal carcinoma on multisequence MRI and output a score that represents the possibility of distant metastases. This would be treated as a treatment decision reference to guide precise treatment of nasopharyngeal carcinoma and bring the dawn to further improve the overall efficacy of nasopharyngeal carcinoma in the future.

## Materials and methods

### Patients

Patients who met the following inclusion criteria between October 2011 and October 2021 at Sichuan Cancer Hospital were selected for this study: 1) patients with pretreatment plain and enhanced magnetic resonance imaging scanning data of nasopharynx and neck; 2) pathology-confirmed nasopharyngeal carcinoma; 3) patients who had finished all the courses of radiotherapy and chemotherapy according to the NCCN Guidelines and institutional standard; 4) patients with regular follow-up at Sichuan Cancer Hospital following treatment; 5) patients who developed single-organ (liver, lung, or bone) metastases more than 6 months after treatment, or patients who live without metastases more than 3 years following treatment.

A total of 85 NPC patients with MSOM and another comparable 101 non-metastasis NPC patients were recruited in this study. Patients were randomly assigned to the training set (N = 140) and validation set (N = 46). Their basic clinical-pathology characteristics, including laboratory tests before treatment, are listed in **Table 1**.

**Table 1:** Clinical and pathological characteristics of patients in this study.

	MSOM	Non-metastases	*p*	Training set	Validation set	*p*
**N**	85	101		140	46	
**Gender**						.539
**Male**	63 (74.1)	72 (71.2)	0.666	100 (71.4)	35 (76.1)	
**Female**	22 (25.9)	29 (28.7)		40 (29.6)	11 (23.9)	
**Age**	47 (27-78)	48 (16-69)	0.459	47 (24-69)	47 (16-68)	0.171
**Target organ**						0.317
**Lung**	25 (29.4)			22 (25.9)	3 (3.5)	
**Liver**	32 (37.6)			24 (28.2)	8 (9.4)	
**Bone**	28 (32.9)			20 (23.5)	8 (9.4)	
**T stage^#^**			0.949			0.326
** T1–2**	18 (21.2	21 (20.8)		27 (19.3)	12 (26.1)	
** T3–4**	67 (78.8)	80 (79.2)		113 (80.7)	34 (73.9)	
**N stage^#^**			0.274			0.056
** N0–2**	56 (65.9)	74 (73.3)		103 (73.6)	27 (58.7)	
** N3**	29 (34.1)	27 (26.7)		37 (26.4)	19 (41.3)	
**Clinical stage^#^**			0.153			0.622
** III**	31 (36.5)	27 (26.7)		45 (32.1)	13 (28.3)	
** IV**	54 (63.5)	74 (73.3)		95 (67.9)	33 (71.7)	
Pathology type^*^			0.902			0.434
** I**	1 (1.2)	1 (1.0)		1 (0.7)	1 (2.1)	
** II**	84 (98.8)	100 (99.0)		139 (99.3)	45 (97.9)	
**PNI**			0.315			0.233
**≥Medial**	40 (47.0)	55 (54.4)		68 (48.6)	27 (58.7)	
** <Medial**	45 (53.0)	46 (54.6)		72 (51.4)	19 (41.3)	
**HGB** ** ≥Medial**	39 (45.9)	55 (45.6)	0.244	67 (47.9)	27 (58.7)	0.202
** <Medial**	46 (54.1)	46 (54.6)		73 (52.1)	19 (41.3)	
**Target therapy**			0.784			0.488
** Yes**	27 (31.8)	34 (33.6)		44 (31.4)	17 (37.0)	
** No**	58 (68.)	67 (66.4)		96 (68.6)	29 (63.0)	
**cDDP**			0.927			0.724
** ≥200 mg/m^2^**	55 (64.7)	66 (65.4)		110 (78.6)	35 (76.1)	
** <200 mg/m^2^**	30 (35.3)	35 (34.6)		30 (21.4)	11 (23.9)	
**Dose of GTV**			0.796			0.648
** ≥70 Gy**	66 (77.6)	80 (79.2)		111 (79.2)	35 (76.1)	
** <70 Gy**	19 (22.4)	21 (20.8)		29 (20.8)	11 (23.9)	

**^#^** Clinical stage was restaged according to the 8th AJCC staging system. *Pathology type, I, keratinizing squamous cell carcinoma; II, non-keratinizing carcinoma; HGB, hemoglobin; PNI, prognostic nutritional index; CDDP, cumulative dose of cisplatin. Chi-square test was used to compare the difference between clinical pathological markers of each group.

### MRI scanning

Patients in this study underwent MRI examination prior to treatment. Head and neck coils with a 1.5-T scanner (Avanto, Siemens, Germany) were used for scanning. All metal objects were not allowed to bring into the scanning room. Motion artifact and magnetic susceptibility were avoided by asking patients, keeping the head and neck fixed without deglutition during scanning. T1WI and T2WI were obtained prior to contrast drug injection. Gadolinium diethylenetriamine penta-acetic acid (Gd-DTPA, 0.1 mmol/kg) was injected to acquire axial fat-suppressed CE-T1WI. All images were reconstructed from the k-space using the inverse Fourier transform with the linear filling method, as we reported previously (21). MRI scanning parameters are listed in **Supplementary Material 1**.

### Image processing

After transferring MRI images into the radiotherapy target volume delineation system MIM Software (Beijing Co., Ltd.), two experienced radiation oncologists with more than 10 years of experience in head and neck cancer delineated gross tumor volume (GTV) and lymph node gross tumor volume (GTVln) in transverse TIWI, T2WI, and CE-T1WI, respectively. When disagreements occurred during the contouring process, a third researcher stepped in to resolve the disagreements by discussions.

For better performance and convenience, we implemented data processing by following a three-step procedure. First, the format of images was converted from DICOM format into JPEG format, and the contours of lesions were transformed into binary masks and coordinates of bounding boxes. Second, both the pixel spacing and slice interval were normalized to 1 mm and the range of pixel values was normalized between 1 and 255. Third, (22) all labels included in this study were transferred to COCO format and all the input images were resized to the same size (512 × 512). All the above three steps were performed in SimpleITK and OpenCV.

### Detection and prediction model building based on multisequence MRI

In order to develop an accurate and robust detection and prediction model based on deep learning methods, a large scale of high-quality annotated data is required. To reduce the limitation of data, we built a new framework to make metastasis prediction for NPC based on transfer learning.

**Figure 1:**
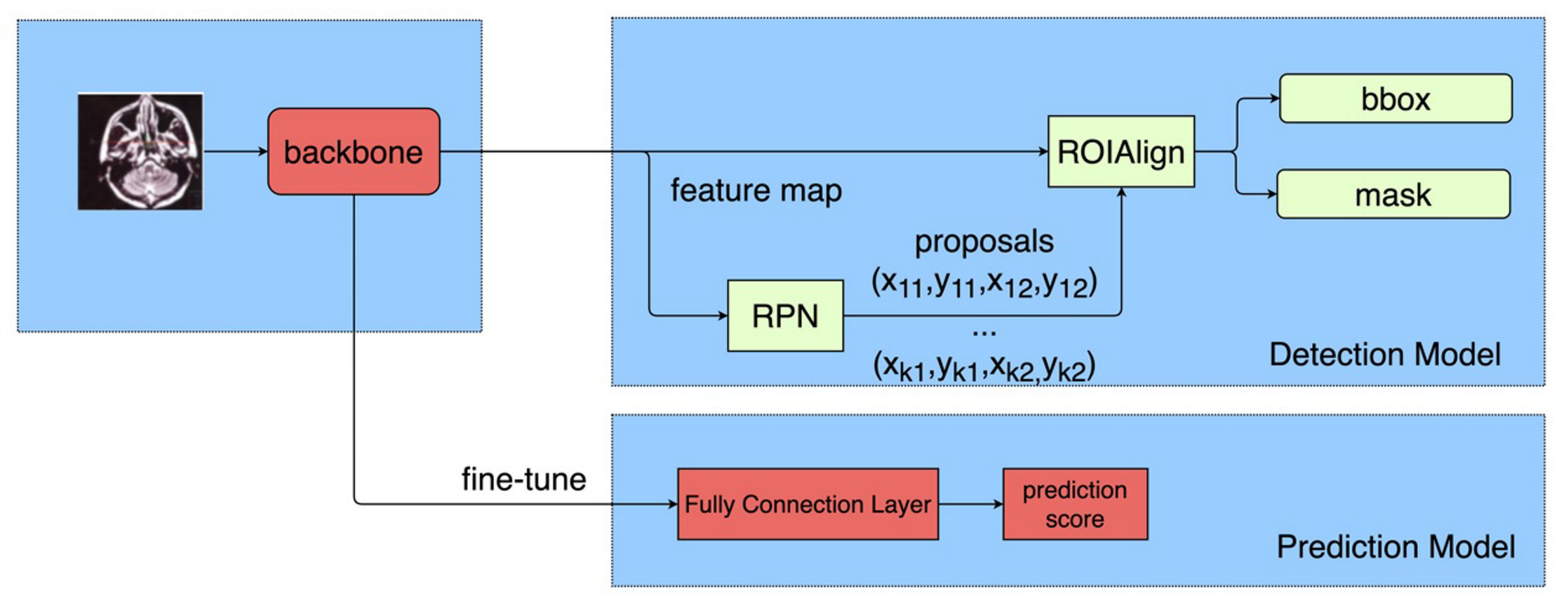
Workflow of deep learning and model building.

Firstly, a detection model was developed to localize and classify GTV and GTVln. During the training stage, the feature extraction module of the detection model could learn low-level location and high-level semantic features of tumors. Also, the model could pretrain the parameters of the feature extraction module, which would be helpful for the convergence of the prediction model in the second stage. The prediction network was fine-tuned separately to make prediction. The feature extraction module of the prediction model was initialized by the common feature extraction module of the detection model in the first stage. There were plenty of instance detection models and feature extraction models in the deep learning field. In this paper, we apply one of the most common detection models, Mask R-CNN (23). The common feature extraction model of the trained model in each stage was ResNet (24) with the Feature Pyramid Network (FPN) (25). The FPN model was proposed to reduce the negative impact of various scales of GTV and GTVln. The overview of the proposed two-stage framework is presented in **Figure 1**. The prediction model would output a score valued between 0 and 1 to represent the possibility of MSOM when the MRI data of a specific patient were inputted.

For single-organ metastasis prediction, a patient was considered as high risk when the average score of all slices that the prediction model outputs was equaled to or higher than the best cutoff value of MRI MSOM score calculated from the ROC curve.

### Metachronous single-organ metastasis prediction model based on MRI and clinical variables

To improve the prediction performance of the model based on deep learning of MRI, we added the clinical data (age, gender, clinical stage, prognostic nutritional index (PNI), hemoglobin (HGB), treatment options, radiation dose, cumulative dose of cisplatin, and cycle of chemotherapy) to the deep learning model based on multisequence MRI. The clinical data and MRI sequence data feature were concatenated to a vector before the last average pool layer and full-connection layer. The combined model was tested in the same validation set to show the ability to predict metachronous single-organ metastases of NPC.

### Statistical analysis

Statistical analyses were performed using SPSS 22.0 statistical software. The Kaplan–Meier method and log-rank text were applied to compare survival. The chi-square test was used to compare the difference between clinical pathological markers of each group. The difference was considered statistically significant when p < 0.05.

### Experimental setup

#### Environment

The programming language Python (Version 3.9.12) was used in Ubuntu 20.04.1 LTS to build automatic tumor segmentation and DSOM prediction models. The details of environment and installation are shown in **Supplementary Material 2**. To improve the reproducibility of the proposed methods, we conducted the experiments based on MMDetection (26), a common open-source detection framework.

#### Implementation details

The Mask R-CNN with default settings was applied to detect GTV and GTVln. The backbone applied was ResNet-50 with FPN. Five stages consisted of ResNet-50. The number of the out channel in each stage was 64, 256, 512, 1,024, and 2,048. It was denoted that C1, C2, C3, C4, and C5 was the output of the last residual block in five stages. In FPN, {C2, C3, C4, C5} was considered as the input to construct feature pyramid structures. The final outputted feature maps were denoted as {P2, P3, P4, P5}, according to {C2, C3, C4, C5}. The number of channels for all these feature maps was 256. For the anchor generator, there were five scales and three aspect ratios for anchors, and the threshold of IoU between the ground truth and anchors was set to 0.5. For the test pipeline, the IoU threshold in the NMS process was set to 0.7 and 0.5, respectively, in RPN and RCNN. All the above parameter settings followed the work by (23).

For the prediction model, the feature extraction model was the same as the backbone of Mask R-CNN. After the feature extraction model, the P3 feature map was inputted into the final module. The size of the feature map was 256 × 32 × 32. The final module consisted of one average pool layer, one full-connection layer, and one sigmoid activation layer. The last output dimension was only one, which denoted the possibility of MSOM.

For the early fusion prediction model, the parameters before the full-connection layer were the same as above. The clinical data were concatenated just before the full-connection layer.

#### Training

Due to the difference of the detection model and prediction model, the detection model was optimized with batch size as 4 and max epoch as 24. The prediction model was optimized with batch size as 2 and max epoch as 20. The detection and prediction model codes are detailed in **Supplementary Material 3**.

## Results

### Characteristic of MSOM from NPC

Of the 85 NPC patients with MSOM (including 32 liver metastases, 25 lung metastases, and 28 bone metastases), the median time to metastases was 13 months (7–36 months) after treatment. The clinical and pathological characteristics of patients in the MSOM and non-metastasis groups and the distribution between the training set and validation set are shown in **Table 1**.

### Performance of tumor detection on multisequence magnetic resonance imaging

To show the performance of the tumor detection model, the common evaluation metrics for object detection models of AP (average precision) and mAP (mean average precision) were used. AP refers to the area under the Precision and Recall curves, whereas mAP represents the average of the AP values of each category. The concerned categories in this study were GTV and GTVln. According to the characteristics of MRI images and tumors, we adopted AP@0.5 and mAP@0.5 to evaluate the performance of the object detection model. It meant that a candidate was considered as a true positive when the Intersection over Union (IoU) overlapped with any ground-truth bounding boxes equal to or higher than 0.5 and considered as a false positive when the IoU value was lower than 0.5. The overall results of the detection model are presented in **Table 2**. The higher the score of AP and mAP is, the better the model performs.

**Table 2:** The performance of Mask R-CNN.

	mAP@0.5	AP@0.5 for GTV	AP@0.5 for GTVln
**T2WI**	52.05	49.4	54.7
**T1WI**	43.25	49.0	37.5
**CE-T1WI**	57.60	59.6	55.6

The above table shows that the performance of the adopted detection model for CE-T1WI images was the best among the three. It may be due to the high quality of images that CE-T1WI performed best to detect GTV and GTVln for the proposed detection model. The mAP@0.5 of the tumor detection model based on CE-T1WI was 57.6, whereas the AP@0.5 of the detection model based on CE-T1WI for GTV was 59.6 and 55.6 for GTVln, which were better than that of the detection model based on TIWI and T2WI (**Table 2**).

**Figure 2:**
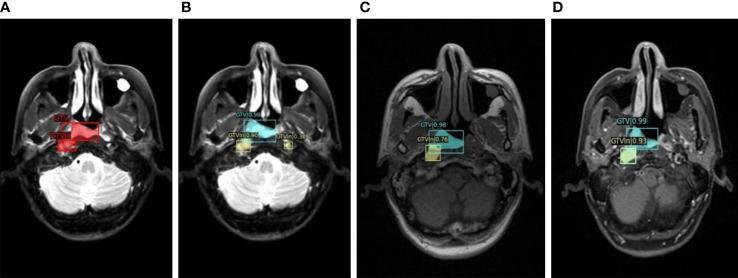
The visualization of detection model for one example slice in three channels. **(A)**. Manually labeled GTV and GTVln on T2WI; **(B)** Automatically detected GTV and GTVln on T2WI; **(C)** Automatically detected GTV and GTVln on T1WI; **(D)** Automatically detected GTV and GTVln on CE-T1WI).

To show the automatic tumor detection result in the multisequence image, the visualization of the detection model for one example slice in three channels is shown in **Figure 2**. As shown in the figure, each candidate predicted by detection models was labeled with bounding boxes (bboxes) and confidence coefficient. In addition, the ground truth was labeled with red bboxes in the first subfigure. The GTV candidates and GTVln candidates were annotated in yellow and blue, respectively.

### Performance of the MSOM prediction model based on MRI and the integrated model based on MRI and clinical variables in the validation set

To evaluate the performance of the prediction model, AUC (area under the curve), sensitivity, recall, and accuracy were adopted. Note that the sensitivity, recall, and accuracy were calculated at the median threshold of the predictive risk scores. To show the advantage of the model we proposed, the validation set in this study was also used to verify the performance of the deep learning model reported by Zhang Lu in 2021 (20).

Based on the T1WI sequence, the precision, recall, accuracy, and AUC of the proposed prediction model were 0.600, 0.600, 0.692, and 0.722 (95% CI, 0.530–0.909). The precision, recall, accuracy, and AUC of the proposed combined prediction model were 0.769, 0.667, 0.795, and 0.738 (95% CI, 0.535–0.926). The precision, recall, accuracy, and AUC of the comparison model were 0.563, 0.643, 0.684, and 0.717 (95% CI 0.543–0.891). By comparing with the model reported by Zhang Lu, we found that the AUC of the model based on T1WI alone was higher than the comparison method by 0.5%, whereas the AUC of the integrated model based on T1WI and clinical variables was higher than the comparison method by 2.1%. The results of experiments based on T1WI are shown in **Table 3**.

**Table 3:** The performance of the proposed prediction model based on MRI alone or MRI integrated with clinical variables in the validation set.

Model	Precision	Recall	Accuracy	AUC (95% CI)
TIWI				
Zhang Lu et al.	0.563	0.643	0.684	0.717 (0.543-0.891)
Ours	0.600	0.600	0.692	0.722 (0.530-0.909)
Ours (fusion model)	0.769	0.667	0.795	0.738 (0.535-0.926)
T2WI				
Zhang Lu et al.	0.556	0.625	0.650	0.685 (0.465-0.817)
Ours	0.727	0.438	0.725	0.695 (0.458-0.849)
Ours (fusion model)	0.750	0.563	0.750	0.719 (0.537-0.900)
CE-T1WI				
Zhang Lu et al.	0.500	0.500	0.611	0.620 (0.419-0.848)
Ours	0.727	0.533	0.730	0.733 (0.557-0.909)
Ours (fusion model)	0.714	0.667	0.757	0.775 (0.606-0.945)

Based on the T2WI sequence, the precision, recall, accuracy, and AUC of the prediction model we proposed were 0.727, 0.438, 0.725, and 0.695 (95% CI, 0.458–0.849). The precision, recall, accuracy, and AUC of the combined prediction model were 0.750, 0.563, 0.750, and 0.719 (95% CI, 0.537–0.900). The precision, recall, accuracy, and AUC of the comparison model were 0.556, 0.625, 0.650, and 0.685 (95% CI 0.465–0.817). By comparing with the model reported by Zhang Lu, we found that the AUC of the model based on T2WI alone was higher than the comparison method by 1.0%, whereas the AUC of the integrated model based on T2WI and clinical variables was higher than the comparison method by 3.4%. The results of experiments based on T1WI are shown in **Table 3**.

Based on CE-T1WI, the precision, recall, accuracy, and AUC of the prediction model were 0.727, 0.533, 0.730, and 0.733 (95% CI, 0.559–0.909). In that order, the precision, recall, accuracy, and AUC of the fusion model were 0.714, 0.667, 0.757, and 0.775(95% CI 0.606–0.945). The precision, recall, accuracy, and AUC of the comparison model were 0.500, 0.500, 0.611, and 0.620 (95% CI 0.419–0.848). By comparing with the model reported by Zhang Lu, we found that the AUC of the model based on CE-T1WI alone was higher than the comparison method by 11.3%, whereas the AUC of the integrated model based on CE-T1WI and clinical variables was higher than the comparison method by 15.5%. The results of experiments based on CE-T1WI are shown in **Table 3**.

**Figure 3:**
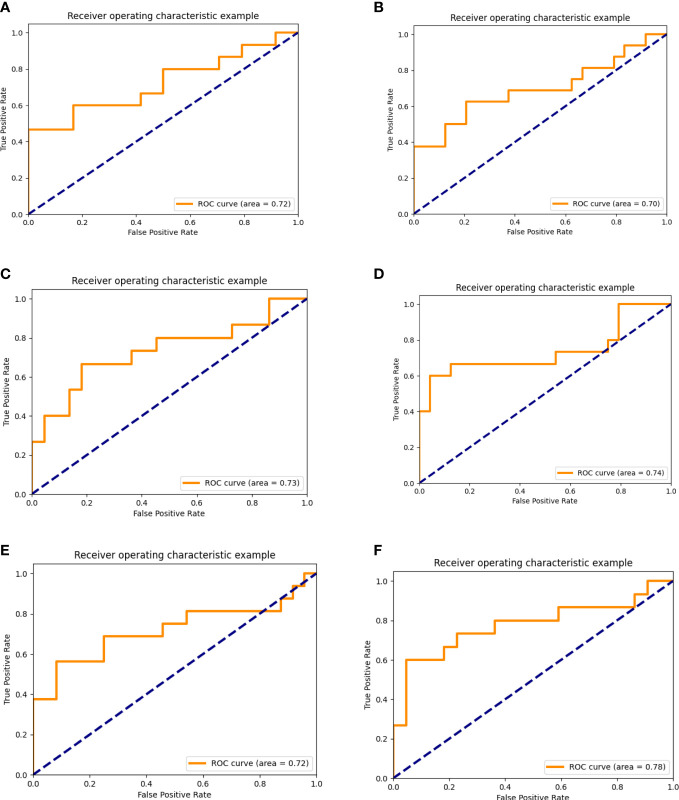
AUC of the prediction model based on multi-sequence MRI alone or integrated with clinical data. **(A–C)**, AUC of the prediction model based on multisequence MRI. **(A)** AUC of the prediction model based on TIWI, **(B)** AUC of the prediction model based on T2WI, **(C)** AUC of the prediction model based on CE-T1WI. **(D–F)**, AUC of the prediction model based on multi-sequence MRI and clinical data. **(D)** AUC of the prediction model based on TIWI and clinical data. **(E)** AUC of the prediction model based on T2WI and clinical data. **(F)** AUC of the prediction model based on CE-T1WI and clinical data).

By comparison, we found that the overall performance of the prediction model based on T1WI and CE-T1WI was quite good in the validation set (AUC >0.7). The accuracy and AUC of the integrated model were better than those of the model based on MRI alone. All the AUC values of the integrated model were bigger than 0.7. The biggest AUC value we acquired in the validation set was the integrated model based on CE-T1WI and clinical variables (AUC = 0.775 (95% CI 0.606–0.945)). The AUC map is shown in **Figure 3**.

### Survival of patients according to MSOM risk based on CE-T1WI and clinical variables

We divided patients into high and low MSOM risk groups according to the prediction models we built with data based on CE-TIW alone or integrated with clinical variables. Then, we compared the metachronous single-organ distant metastasis-free survival (DMFS) and overall survival (OS) of patients with high and low MSOM risks.

According to the model based on CE-T1WI, the 3-year metachronous single-organ DMFS of patients in the high and low MSOM risk groups were 10.8% and 95%, respectively (p < 0.001, X^2^ = 166.06). The 3-year OS of patients in the high and low MSOM risk groups were 85.1% and 97%, respectively (p < 0.001, X^2^ = 10.49)).

**Figure 4:**
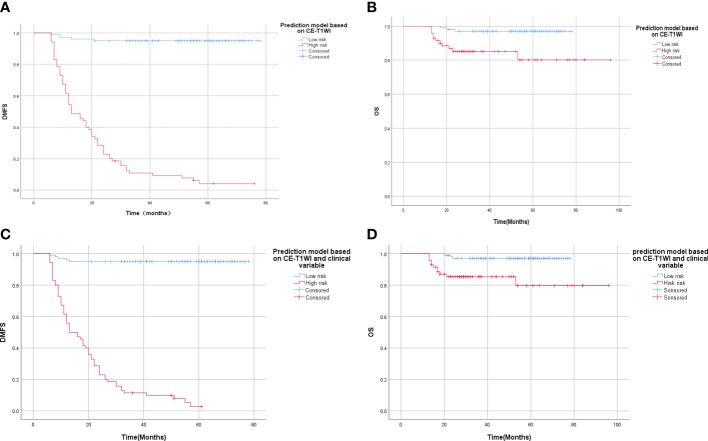
Survival curve of patients with high and low MSOM risk. **(A)** Distant metastasis-free survival (DMFS) of patients in different risk groups according to the CE-T1WI-based model. **(B)** Overall survival (OS) of patients in different risk groups according to the CE-T1WI-based model. **(C)** Distant metastasis-free survival (DMFS) of patients in different risk groups according to integrated model. **(D)** Overall survival (OS) of patients in different risk groups according to the integrated model).

According to the integrated model based on CE-T1WI and clinical variables, the 3-year metachronous single-organ DMFS of patients in the high MSOM risk and low MSOM risk groups were 11.4% and 95%, respectively (*p* < 0.001, X^2^ = 164.29). The 3-year OS of patients in the high and low MSOM risk groups were 85.3% and 97%, respectively (*p* = 0.001, X^2^ = 10.69). Patients with the low MSOM risk would achieve better DMFS and OS than those with the high MSOM risk in both of the model (**Figure 4**).

## Discussion

MRI is a standard examination technique with outstanding image resolution; MR-based radiomics can provide diagnostic, prognostic, or predictive information related to NPC that cannot be observed with the naked eyes, and it has shown great potential clinical application in tumor staging, image guiding, prognosis prediction, and treatment decision (27–29). With the advent and development of medical big data, the combined application of computer and machine learning methods makes the application of MR-based radiomics in nasopharyngeal carcinoma more promising (30–33). With the inherent advantages of high soft tissue resolution and multisequence imaging, MRI showed unique advantages over CT or PET-CT, in the diagnosis and treatment of newly treated nasopharyngeal carcinoma (34, 35). Nasopharynx and neck MRI scanning has become an essential and also important pretreatment evaluation approach, which was suggested in the guidelines and was widely used in clinical activities (36, 37). Lee S et al. evaluated the prognostic value of magnetic resonance imaging (MRI)-based radiomics for newly diagnosed NPC in a systematic review and meta-analysis, which showed that MRI-based radiomics revealed an overall modest prognostic value in predicting PFS (mean C-index, 0.76; 95% CI, 0.69–0.84) (28). Wu G et al. confirmed that dynamic contrast-enhanced MRI predicts PTEN protein expression, which can function as a prognostic measure of progression-free survival in NPC patients (38). Zhang Lu et al. developed a distant metastasis MRI-based model (DMMM), which showed an AUC of 0·792(95% CI, 0·633–0·952) in validation cohorts (7). Different from past studies which used the traditional radiomics methods, we developed an automatic tumor detection and segmentation approach based on deep learning to predict MSOM, which exhibited similar prediction ability (AUC = 0.775).

Clinical staging is currently the most important tool to predict the prognosis of NPC. However, the accuracy of the model based on the N stage to predict distant metastases was only about 57%. Another shortcoming is that it falls short of reflecting the heterogeneity of individual tumors (6). Several studies have reported that lymph node gross tumor volume (GTVln), gross tumor volume of the nasopharynx (GTVnx), circulating CD4 T lymphocytes, lactate dehydrogenase, lactate dehydrogenase (LDH) level before treatment, hemoglobin level, and EBV DNA level were significantly associated with the distant metastases of NPC (6, 39, 40). Several studies have shown that the plasma EBV DNA level before treatment was related to the clinical stage and tumor burden of nasopharyngeal carcinoma, and it was currently considered to be the most important molecular marker for complementary clinical staging (41, 42). Variations in EBV DNA testing in different laboratories and in endemic and non-endemic areas limit its clinical application, and there is currently a lack of a recognized cutoff value between low- and high-risk patients (43). Several studies have explored gene expression-based signature to predict distant metastases (6, 44, 45). However, the hefty cost in gene testing limited its clinical application, although its accuracy rate reached about 75%.

When it comes to deep learning utilized in this study, it has its intrinsic advantages by avoiding feature engineering, lowering barriers to entry and sharing knowledge across domains (46). Instead of manually designing rules, deep learning can optimize the lost function as much as possible to learn the rules. Moreover, the potential features of the data can be mined as much as possible. To sum up, deep learning belongs to end-to-end learning, and the results can be obtained by inputting data. This is both convenient and fast (30, 46). Compared with hand-crafted radiomics methods, the deep-learning model is relatively easy to operate because it only requires inputting the MR images to end-to-end output a predictive value (20). Wang et al. also suggested that a deep-learning model showed better performance than conventional radiomic and clinical models (47). In this study, we use MMDetection, an object detection toolbox that contains a rich set of object detection and instance segmentation methods as well as related components and modules, to improve the reproducibility of detection (26). With modular design and high efficiency, this toolbox supports multiple frameworks out of box and finally improve repeatability and reproducibility of the module.

The concept of metachronous metastases was initially applied in patients with colorectal cancer who suffered from liver metastases after treatment (48). Oligometastases was defined as metastases that are limited in both number (usually, less than 5) and location (49, 50). It represents a state that could achieve curative outcome or relatively better local control, which eventually transferred to survival benefit by definitive treatment (51). It is well known that oligometastatic state, which usually determines a cancer patient’s final destination, is an inevitable stage toward polymetastases (52). However, no practical and effective markers and systems were applied to predict oligometastases, as oligometastases is only a state from the prospect of treatment and survival. Single location or target organ metastasis seems to be a better representation of an intermediate state of the disease in the view of tumor progression. Patients with single-organ metastases usually showed its peculiar biological profile and clinical characteristics (53, 54). Although the risk of metastases and omics characteristics of the same tumor in different target organs are different, patients who developed single-organ metastases after treatment might share somewhat common genetic and radiomic characteristics that attributed to its intrinsic tumor heterogeneity (53).

Early detection of patients with high risk to develop MSOM prior to treatment can provide relatively sufficient information about the heterogeneity of tumors, which can guide an individualized treatment plan. We compared the single-organ DMFS and OS of patients with high and low MSOM risks and found that patients with a high risk of MSOM had lower 3-year single-organ DMFS (10.8% vs. 95%, *p* < 0.001) and OS (85.1% vs. 97%) in the CE-T1WI-based prediction model. For patients with a low risk of MSOM, it is possible that concurrent chemoradiotherapy would be good enough, as those patients benefit less from neoadjuvant or adjuvant or targeted therapy. For patients with high risk of MSOM, more aggressive treatment strategies should be given. As reported in A phase 3, multicenter, randomized controlled trial, patients with high-risk locoregionally advanced NPC who received metronomic capecitabine could achieve better failure-free survival (55). In this sense, this study provided a new automatic approach to select patients who might benefit from aggressive treatment. Different from other studies to explore distant metastases (including synchronous and metachronous, multi- and single-organ metastases) of NPC (20, 44–46), this study, as far as we know, was the first one to explore MSOM based on multisequence MRI and deep learning.

Although this study exhibits good performance of the MSOM prediction model based on multisequence MRI and deep learning, it is by no means flawless. Firstly, since this is a retrospective study conducted in one research center, it comes with some intrinsic limitations, such as consistency of the enrolled patients and uniformity of the treatment plan. Secondly, due to the long-time span of the enrolled patients, the data in our study were produced by the MRI image scanning machines with somewhat diversified scanning parameters. Although we performed image quality assessment and standardized processing to reduce the image variations, they cannot be considered to be adequately uniform and synchronically comparable. In consideration of the abovementioned deficiencies, further external validation conducted in different research centers is thus beckoned. In this sense, the shortcomings of this study could transform into invitation for follow-up studies.

The main innovations and contributions of this study are as follows. 1) The concept of metachronous single-organ metastases of nasopharyngeal carcinoma was proposed for the first time. 2) We are the first to propose a novel two-stage framework based on transfer learning to make prediction for single-organ metastases of NPC. 3) A prediction model based on CE-T1WI alone or combined with clinical indicators was proposed to achieve better prediction performance. 4) It provides an important reference for accurate diagnosis, treatment, and prediction of nasopharyngeal carcinoma and has important clinical application value. The main research direction in the future is to build a prospective study queue, explore and verify the MSOM intelligent prediction model, and carry out clinical studies based on the MSOM intelligent prediction model. For high-risk patients, the risk of distant metastases would be reduced and the overall curative outcome would be improved by adjusting chemotherapy intensity and maintenance treatment strategy; for low-risk patients, the treatment intensity might be reduced to alleviate therapeutic response in such patients.

In conclusion, we proposed and built an automatic tumor detection and segmentation approach to predict metachronous single-organ metastases of NPC based on MRI and deep learning; The overall performance of the model was quite good, and further studies to validate and applicate its clinical value are warranted.

## Data availability statement

The data analyzed in this study is subject to the following licenses/restrictions: Please contact the corresponding author for data requests. Requests to access these datasets should be directed to langjy610@163.com.

## Ethics statement

The study was approved by the insititutional review board of Sichuan Cancer Hospital(SCCHEC-02-2022-160). As this is an observational study, the Sichuan Cancer Hospital Research Ethics Committee decided to waive the requirement to get informed consent.

## Author contributions

JL, YR, and GX designed this study. YH, YZ, and QY conducted the study and analyzed the results, developed the model, and drafted the manuscript under the supervision of JL, YR, and GX. YL, PZ, JR took part in the drawing target outline, data extraction, and development of the model. YH took part in the research general design, data extraction, and development of the model. JL, YR, and GX have contributed equally to this work and share corresponding authorship. The remaining authors are ranked by their contribution to research. All authors contributed to the article and approved the submitted version.
