# Supplementary material for: Automatic tumor segmentation and metachronous single-organ metastasis prediction of nasopharyngeal carcinoma patients based on multi-sequence magnetic resonance imaging
Source: Front Oncol. 2023 Mar 28;13:953893. doi: 10.3389/fonc.2023.953893 (PMC10099248; doi:10.3389/fonc.2023.953893)
Supplement: Supplementary file 1 [file DataSheet_1.docx]

**1.1 Acquisition parameters**

The 1.5T MRI acquisition parameters were as follows: (1) axial T2-weighted spin-echo images, repetition time (TR)/echo time (TE) = 3280-8290 ms/72-95 ms, slice thickness = 3-4 mm, spacing between slices = 3.6-7.44 mm, echo train length (ETL) = 13-14, number of excitation (NEX) = 1-3, flip angle (FA) = 150^°^, acquisition type = 2D, in-plane resolution = 0.33 × 0.33 mm^2^ to 0.78 × 0.78 mm^2^; (2) axial contrast-enhanced T1-weighted spin-echo images, TR/TE = 610-1090 ms/11-19 ms, slice thickness = 3 mm, spacing between slices = 3.3-4.5 mm, ETL = 2-3, NEX = 1-2, FA = 138-150^°^, acquisition type = 2D, in-plane resolution = 0.39 × 0.39 mm^2^ to 1.17 × 1.17 mm^2^ ; (3) axial contrast- enhanced T1-weighted gradient-echo images, TR/TE = 306 ms/4.76 ms, slice thickness = 3 mm, spacing between slices = 3.3 mm, ETL = 1, NEX = 1, FA = 80^°^, acquisition type = 2D, in-plane resolution = 0.46 × 0.46 mm^2^; (4) axial contrast-enhanced T1-weighted gradient-echo images, TR/TE: 5.95 ms/2.76 ms, slice thickness = 3 mm, spacing between slices = 0 mm, ETL = 1, NEX = 1, FA =10^°^, acquisition type = 3D, in-plane resolution = 0.98 × 0.98 mm^2^.
