# Supplementary material for: Automatic tumor segmentation and metachronous single-organ metastasis prediction of nasopharyngeal carcinoma patients based on multi-sequence magnetic resonance imaging
Source: Front Oncol. 2023 Mar 28;13:953893. doi: 10.3389/fonc.2023.953893 (PMC10099248; doi:10.3389/fonc.2023.953893)
Supplement: Supplementary file 2 [file DataSheet_2.doc]

## Enviroment and Installation

```

Operating System: Ubuntu 20.04.1 LTS

Python: 3.9.12

GPU : NVIDIA GeForce RTX 3070

NVCC: Build cuda_11.0_bu.TC445_37.28540450_0

GCC: gcc (Ubuntu 7.5.0-6ubuntu2) 7.5.0

PyTorch: 1.10.0

PyTorch compiling details: PyTorch built with:

- GCC 7.3

- C++ Version: 201402

- CUDA Runtime 11.3

MMDetection: 2.16.0

```

You can run the script install.sh to quickly install the enviroment.

## Introduction

- Prepare data: To prepare the data included in this study, you can run the main.py in 'Data' folder.

- Model: The codes in 'Model' folder is built based on [MMDetection](https://github.com/open-mmlab/mmdetection/tree/v2.16.0), the detail tutorial of MMDetection can reference to [official tutorials](https://github.com/open-mmlab/mmdetection/tree/v2.16.0/docs/tutorials).

The tiny modifition is in mmdet/datasets/coco.py,mmdet/datasets/custom.py and mmdet/core/evaluation. You can modify the config files in 'configs/' folder to adapt your own enviroment and settings. Note that every time you want to modify the codes in mmdet/, you must delete the 'build'、'dist' and 'mmdet.egg-info' folders and run `python setup.py install --user`
